# Air pollution exposure disparities in US public housing developments

**DOI:** 10.1038/s41598-022-13942-3

**Published:** 2022-06-14

**Authors:** Jayajit Chakraborty, Timothy W. Collins, Sara E. Grineski, Jacob J. Aun

**Affiliations:** 1 Department of Sociology and Anthropology, University of Texas at El Paso, El Paso, TX USA; 2 Department of Geography, University of Utah, Salt Lake City, UT USA; 3 Department of Sociology, University of Utah, Salt Lake City, UT USA

**Keywords:** Environmental impact, Environmental sciences, Environmental social sciences

## Abstract

Fine particulate matter 2.5 microns or less in diameter (PM_2.5_) is widely recognized to be a major public health concern. While ethnic/racial minority and lower socioeconomic status individuals in the US experience higher PM_2.5_ exposure, previous research on social disparities in PM_2.5_ exposure has not examined residents of federally-assisted public housing developments (PHDs). Here we present the first national-scale analysis of the relationship between outdoor PM_2.5_ exposure and PHD residency in the US, as well as exposure disparities within the population of households residing in PHDs. We integrated data on average annual PM_2.5_ concentrations (2011–2015) with US Department of Housing and Urban Development data on PHDs (2015), and socio-demographic information from the 2011–2015 American Community Survey. Results from multivariable generalized estimating equations indicated that PHD locations, units, and residents are significantly overrepresented in neighborhoods with greater PM_2.5_ exposure, after accounting for clustering, urbanization, and other socio-demographic factors. Additionally, significantly higher percentages of Black, Hispanic, disabled, and extremely low-income households reside in PHDs with greater PM_2.5_ exposure. Findings represent an important starting point for future research and emphasize the urgent need to identify gaps in environmental, public health, and housing policies that contribute to disproportionate air pollution exposures among PHD residents.

## Introduction

Fine particulate matter 2.5 microns or less in diameter (PM_2.5_) is widely recognized to be a major public health concern^1,2^ that has been linked to multiple adverse health outcomes, including mortality and morbidity due to cardiovascular disease, respiratory problems, diabetes, neurodegenerative disease, and others^3–9^. In the US, PM_2.5_ exposure is a significant health risk factor responsible for 63% of all deaths from environmental causes and 3% of deaths from all causes^10^. Evidence suggests that ethnic/racial minorities and individuals of lower socioeconomic status in the US face a higher risk of death from PM_2.5_ exposure^11,12^. Studies on the spatial distribution of PM_2.5_ in the US have also documented disproportionately higher levels of exposure for these socially disadvantaged groups^13–21^.

While there is a growing scholarly and policy focus on social disparities in ambient PM_2.5_ exposure, there has been no empirical analysis focused on residents of federally subsidized rental housing, in general, and public housing developments (PHDs), in particular. Households residing in PHDs represent a particularly vulnerable subgroup because public housing in the US is only available to households with incomes at 30% of the Area Median Income or less. As a consequence, PHDs often contain higher proportions of ethnic/racial minorities and individuals in poverty compared to the general population within a given jurisdiction^22^. PHDs are also inhabited by other vulnerable populations such as disabled and elderly residents, people with pre-existing health conditions, and others with limited capacity or resources for addressing the adverse health impacts of air pollution exposure. Historically, public housing projects in the US were located in areas of lower economic value, communities with limited local resistance to these projects, and undesirable neighborhoods that are adjacent to industrial facilities, major transportation corridors, and environmentally contaminated sites^23,24^.

To our knowledge, only two studies have investigated the relationship between the location of environmental pollution sources and federally subsidized housing projects in the US. Cutter et al.^23^ analyzed eight medium-sized US metropolitan areas and found families in federally assisted public housing to have greater risk potential from hazardous facilities, based on both proximity and reported toxic emissions. A more recent study found 70% of hazardous Superfund sites listed on the US Environmental Protection Agency (EPA)’s National Priorities List were located with one mile of federally assisted housing developments^24^. While these studies did not examine PM_2.5_ exposure, they highlight the need for additional and more detailed analysis of social disparities in the spatial distribution of ambient air pollution for public housing projects. With more than 1.2 million US households living in PHDs^24^, more systematic research is urgently needed to determine if PHDs and their residents are disproportionately exposed to higher air pollution levels and specifically, higher concentrations of PM_2.5_.

Our study responds to this need by presenting the first national-scale assessment of the relationship between outdoor exposure to fine particulate air pollution and PHD residency, as well as exposure disparities within the population of households residing in PHDs, in the US. This research has two related objectives. First, we seek to determine whether PHD locations, units, and residents, respectively, are significantly overrepresented in neighborhoods burdened by higher outdoor PM_2.5_ concentrations, after controlling for spatial clustering and relevant socio-demographic factors. Following this neighborhood level analysis, we investigate ethnic/racial and socioeconomic inequalities within PHDs. This second objective thus focuses on determining whether outdoor PM_2.5_ concentrations are significantly greater around locations of PHDs where higher percentages of socially disadvantaged households reside. We conduct analyses at the census tract-level in counties containing public housing to address our first research objective and at the PHD-level to address our second objective, with the continental US (lower 48 states and Washington DC) representing our study area.

For this study, we combined high-resolution data on the average annual concentrations of ambient PM_2.5_ developed by the Center for Air, Climate and Energy Solutions^25^ with US Department of Housing and Urban Development (HUD) data^26^ for PHDs, and census tract-level socio-demographic information from the American Community Survey (ACS). Our statistical analyses are based on multivariable generalized estimating equations (GEEs) that account for spatial clustering of census tracts and locations of PHDs, respectively.

## Results

**Figure 1:**
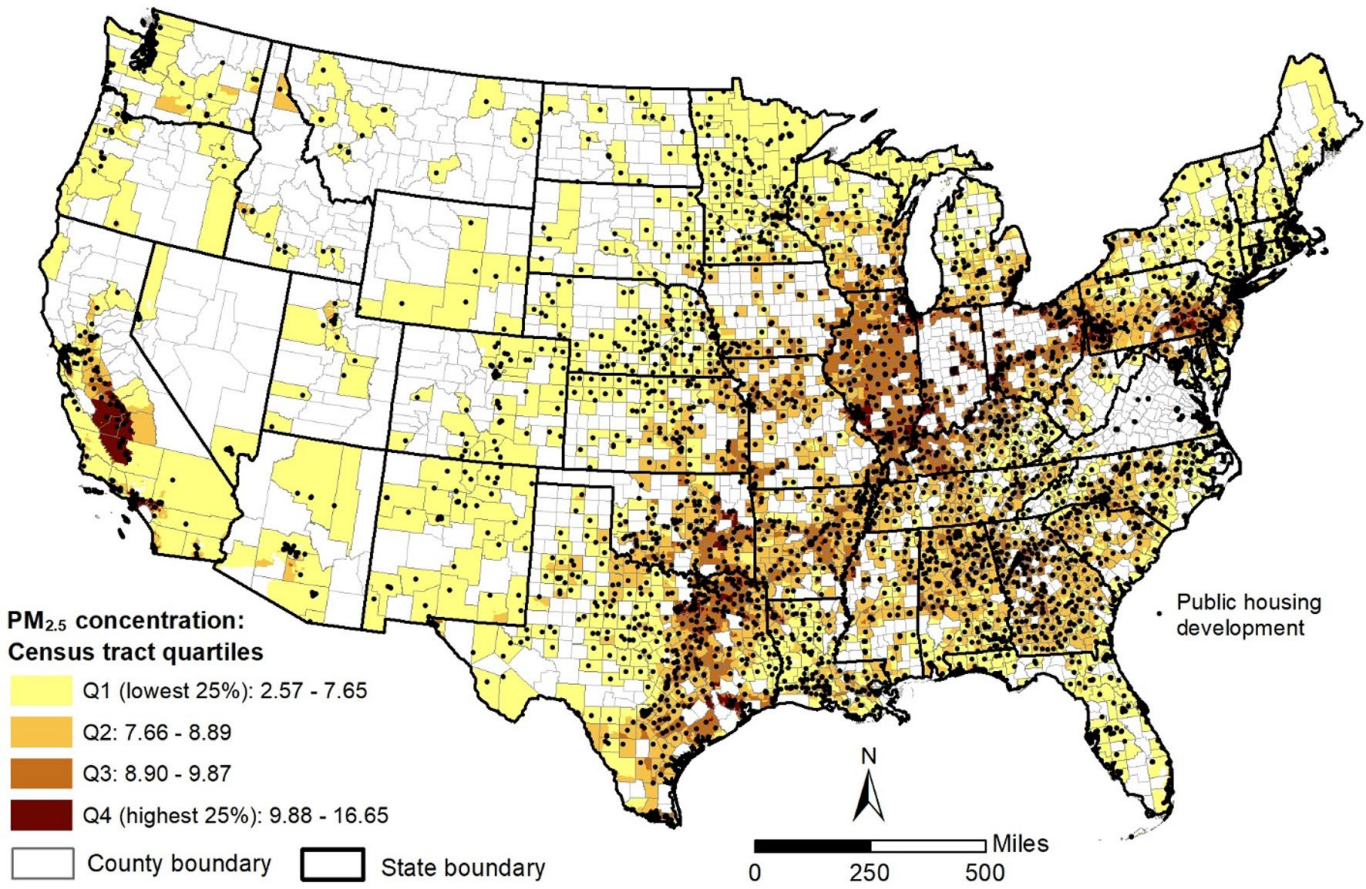
Census tracts in continental US counties with public housing by annual average particulate matter (PM_2.5_) concentration in micrograms per cubic meter (μg/m^3^), 2011–2015. Note: This map was created using *ArcGIS Desktop 10.8.1* (ArcMap) software: https://www.esri.com/en-us/arcgis/products/arcgis-desktop/overview.

The spatial distribution of PM_2.5_ exposure and PHD locations in the continental US are depicted in Fig. 1. This map groups census tracts in counties with public housing into four quartiles based on the 2011–2015 annual average PM_2.5_ concentrations. A large percentage of tracts in the highest quartile (top 25%) of PM_2.5_ exposure are concentrated within metropolitan areas of Illinois, Pennsylvania, Texas, and California. Tracts in the lowest quartile (bottom 25%), in contrast, are dispersed across states of the US Midwest and within Florida.

**Figure 2:**
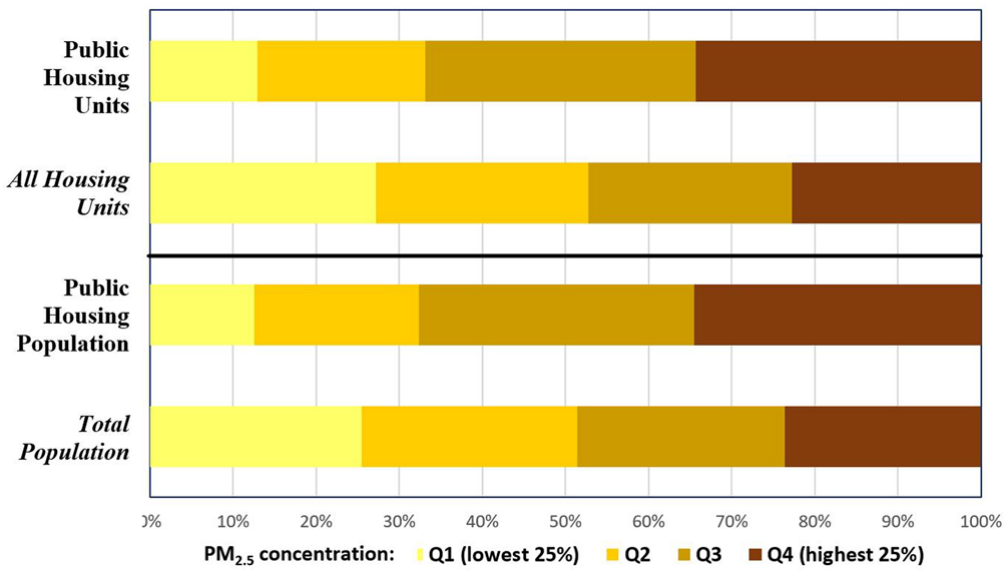
Distribution of housing units and population in the continental US by census tract quartile of PM_2.5_ concentration. Note: In each quartile, annual average particulate matter (PM_2.5_) concentrations in micrograms per cubic meter (μg/m^3^) for 2011–2015 are 2.57–7.65 (Q1), 7.66–8.89 (Q2), 8.90–9.87 (Q3) and 9.88–16.65 (Q4).

Before conducting multivariable analysis, we examined the distribution of PHDs across the four quartiles of PM_2.5_ exposure shown in Fig. 1. For this purpose, we first identified the PHDs located within census tracts in each of the four quartiles of PM_2.5_ concentration. The number of housing units and residents in each of these four groups of PHDs were then summed to derive the PHD unit and population totals for each PM_2.5_ concentration quartile. Figure 2 depicts the number of units and people residing in PHDs within each quartile (as a percentage of each total) and compares them to the corresponding percentages of all housing units and total population in each quartile of PM_2.5_ exposure. About 25% of all housing units and 24% of the total population in the continental US reside within tracts in the lowest quartile (Q1) of PM_2.5_ exposure. However, only about 13% of PHD units and 12% of PHD population, respectively, are located within this set of tracts (Q1). Similar disparities were observed among tracts in the highest quartile of PM_2.5_ exposure. This set of tracts (Q4) encompasses only about 23% of all housing units and 24% of the total population, compared to approximately 34% of PHD units and 35% of the PHD population, respectively. These results thus indicate an overrepresentation of both PHD units and their residents in tracts with highest PM_2.5_ exposure (top 25%), and an underrepresentation of PHD units and residents in tracts facing lowest exposure (bottom 25%). A similar overrepresentation of PHD units and population was observed in the top 50% of tracts based on PM_2.5_ exposure, when tracts in the two highest quartiles (Q3 and Q4) are combined. This set of tracts (top 50%) contains 67% of PHD units and PHD residents, respectively, compared to only about 49% of all housing units and 47% of the total population.

The names, definitions, and summary statistics for all variables used in our tract-level multivariable GEE models are listed in Table 1. Results from GEEs summarizing the association between tract-level PM_2.5_ concentrations and PHD indicators (presence, units, and population) are presented in Table 2. After controlling for spatial clustering of tracts and socio-demographic factors known to be associated with PM_2.5_ exposure in the US (see Methods for further details), the annual average PM_2.5_ concentration indicates a statistically significant and positive relationship (*p* < 0.05) with the presence of PHDs (Model A), percentage of housing units that are in PHDs (Model B), and the percentage of population residing in PHDs (Model C). With regards to the socio-demographic, urbanization, and housing variables used as controls, all three GEE models indicate similar statistical associations. Specifically, PM_2.5_ concentrations are significantly greater (*p* < 0.05) in tracts characterized by higher percentages of non-Hispanic Black residents, people with disabilities, female-headed households, and individuals below poverty, but lower in tracts with higher percentages of people aged 62 or more years and single-family homes. Percent below poverty level also indicates a positive association with PM_2.5_ concentration (*p* < 0.10), but this relationship was not significant (*p* > 0.05). Tracts in both metropolitan and micropolitan areas are exposed to significantly higher average PM_2.5_ concentrations (*p* < 0.001) than those in rural areas.

**Table 1:** Census tract level analysis: variable definitions and summary statistics.

Variables	Definition	Min	Max	Mean	SD
PM_2.5_ pollution exposure (dependent variable)	Average annual concentration of PM_2.5_ (2011–2015) in the tract, in micrograms per cubic meter (μg/m3)	2.57	16.65	8.76	1.66
Presence of PHDs (public housing developments)	Whether or not a PHD located within the census tract (1/0)	0.00	1.00	0.09	n/a
Percent housing units in PHDs	100 * number of units in PHDs divided by total number of housing units in the tract	0.00	100.00	1.06	5.60
Percent population in PHDs	100 * people residing in PHDs within tract divided by total tract population	0.00	100.00	0.95	5.25
Percent Hispanic	% of tract population identifying as Hispanic (ethnicity)	0.00	100.00	16.85	22.04
Percent non-Hispanic Black	% of tract population identifying as non-Hispanic (ethnicity) and Black (race)	0.00	100.00	14.56	22.76
Percent other minority	% of tract population identifying as non-Hispanic (ethnicity) and not White, Black, or Asian (race)	0.00	99.80	7.67	9.97
Percent with disability	% of the civilian non-institutionalized population in the tract reporting any disability	0.00	68.60	13.00	5.63
Percent female headed households	% of households with no adult male members present	0.00	98.50	14.08	8.85
Percent aged 62 or higher	% of tract population aged 62 or more years	0.00	96.40	17.97	8.47
Percent below poverty level	% of individuals in tract with an annual income below the federal poverty level	0.00	96.34	16.98	12.89
Metropolitan	Tracts with Rural–Urban Commuting Area (RUCA) Codes: 1–3 (metropolitan area)	0.00	1.00	0.86	n/a
Micropolitan	Tracts with RUCA Codes: 4–6 (micropolitan area)	0.00	1.00	0.08	n/a
Percent housing units single-family units	Percentage of housing units in tract classified as single-family detached homes	0.00	100.00	60.84	27.24

N = 61,117 tracts (with at least 500 people and 200 housing units) in counties of continental US with public housing.

**Table 2:** Multivariable generalized estimating equations (GEEs) for predicting census tract PM_2.5_ concentrations.

Variables	Model A			Model B			Model C		
	Coeff	SE	*p *value	Coeff	SE	*p *value	Coeff	SE	*p *value
Presence of PHDs	0.162	0.023	< 0.001						
Percent housing units in PHDs				0.026	0.007	< 0.001			
Percent population in PHDs							0.002	0.006	0.002
Percent Hispanic	0.042	0.028	0.132	0.041	0.028	0.148	0.041	0.028	0.151
Percent non-Hispanic Black	0.192	0.020	< 0.001	0.193	0.020	< 0.001	0.193	0.021	< 0.001
Percent other minority	0.001	0.024	0.951	0.001	0.024	0.965	0.001	0.024	0.966
Percent with disability	0.030	0.012	0.015	0.033	0.012	0.007	0.034	0.012	0.006
Percent female headed households	0.132	0.018	< 0.001	0.133	0.018	< 0.001	0.134	0.018	< 0.001
Percent aged 62 or higher	− 0.208	0.015	< 0.001	− 0.209	0.015	< 0.001	− 0.209	0.015	< 0.001
Percent below poverty level	0.039	0.022	0.076	0.041	0.023	0.077	0.042	0.023	0.068
Metropolitan	0.799	0.048	< 0.001	0.769	0.047	< 0.001	0.769	0.047	< 0.001
Micropolitan	0.436	0.046	< 0.001	0.416	0.046	< 0.001	0.416	0.046	< 0.001
Percent housing units single-family	0.008	0.022	0.730	0.008	0.023	0.734	0.007	0.023	0.751
Intercept	7.900	0.044	< 0.001	7.944	0.043	< 0.001	7.944	0.043	< 0.001

N = 61,117 tracts in counties of continental US with public housing. Model specifications are gamma with identity link with an unstructured correlation matrix with controls for clustering in terms of county by median decade of housing stock (7492 clusters). SE is standard error and all *p* values are based on Wald Chi-Square test. Independent variables were standardized.

The names, definitions, and summary statistics for all variables used in our PHD-level multivariable GEE are listed in Table 3. Results from the GEE summarizing the association between annual average PM_2.5_ concentrations and socio-demographic characteristics of households residing in public housing are presented in Table 4. After controlling for spatial clustering of PHDs based on their city of location, we found positive and significant relationships (*p* < 0.05) with the annual average PM_2.5_ concentration for the percentages of PHD households headed by Hispanic and non-Hispanic Black residents, as well as the percentages of people with disabilities and extremely low-income households. The average PM_2.5_ concentration is positively related to the percentages of PHD households headed by females and those aged 62 or more years (*p* < 0.10) , but not significantly (*p* > 0.05). The average PM_2.5_ concentration is also significantly higher around PHDs located in metropolitan and micropolitan tracts, and in tracts with a higher percentage of single-family homes (*p* < 0.01).

**Table 3:** Public housing development level analysis: Variable definitions and summary statistics.

Variables	Definition	Min	Max	Mean	SD
PM_2.5_ pollution exposure (dependent variable)	Average annual concentration of PM_2.5_ (2011–2015) in block group where public housing is located, in micrograms per cubic meter (μg/m^3^)	3.76	16.45	9.07	1.39
Percent Hispanic	% of households: ethnicity of the head of household is Hispanic	0.00	100.00	13.36	23.03
Percent non-Hispanic Black	% of households: race of head of household is Black and ethnicity is not Hispanic	0.00	100.00	44.33	37.59
Percent other minority	% of households: race of the head of household is not White or Black, and ethnicity not Hispanic	0.00	95.00	2.61	7.39
Percent with a disability	% of all persons in public housing households who have a disability	0.00	100.00	26.27	20.73
Percent female headed households	% of households headed by a female resident	2.00	100.00	74.26	14.69
Percent aged 62 or more years headed households	% of households in which the older of the household head or spouse is age 62 or older	0.00	100.00	32.90	25.77
Percent extremely low income households	% of households: income below 30% of local area median family income, as defined by HUD, adjusted for household size	5.00	100.00	67.28	16.42
Metropolitan	Whether or not PHD is located in a census tract with RUCA Codes 1–3 (metropolitan area)	0.00	1.00	0.70	n/a
Micropolitan	Whether or not PHD is located in a census tract with RUCA Codes 4–6 (micropolitan area)	0.00	1.00	0.13	n/a
Percent housing single-family units	Percentage of housing units in tract classified as single-family detached homes	0.00	98.87	50.21	26.52

N = 6685 PHDs with complete data for all independent variables.

**Table 4:** Multivariable GEEs for predicting PM_2.5_ concentration around public housing developments.

Variables	Coeff	SE	*p *value
Percent Hispanic	0.056	0.023	0.012
Percent non-Hispanic Black	0.176	0.020	< 0.001
Percent other minority	0.001	0.011	0.962
Percent with a disability	0.027	0.011	0.018
Percent female headed households	0.019	0.011	0.075
Percent aged 62 or more years headed households	0.018	0.011	0.084
Percent extremely low income households	0.034	0.012	0.004
Metropolitan	0.554	0.103	< 0.001
Micropolitan	0.501	0.093	< 0.001
Percent housing single-family units	0.049	0.017	0.004
Intercept	8.538	0.071	< 0.001

N = 6685 PHDs with complete data for all independent variables. Model specifications are gamma with identity link with an exchangeable correlation matrix with controls for clustering in terms of city in which PHD is located (3337 clusters). SE is standard error and all *p* values are based on Wald Chi-Square test. Independent variables were standardized.

## Discussion

This study sought to extend research on social disparities in the distribution of air pollution by focusing on exposure to fine particulate air pollution for federally subsidized PHDs in the US. Our first research objective focused on determining whether PHD locations, units, and residents are significantly overrepresented in neighborhoods burdened by significantly greater exposure to outdoor PM_2.5_. Our analysis indicated that the presence of PHDs, as well as the overall percentages of both PHD units and residents are significantly greater in neighborhoods with greater annual average PM_2.5_ concentrations, after accounting for spatial clustering, urbanization level, and the presence of socially disadvantaged groups. This spatial concentration of PHD units and residents in more polluted census tracts cannot be explained solely by their disproportionate location in segregated neighborhoods containing significantly higher percentages of ethnic/racial minority and lower socioeconomic status residents, since these variables were used as controls in our statistical models. Our results for the non-Hispanic Black percentage align with previous studies that reported significant racial inequities in the distribution of fine particulate pollution in the US^17–21,27–29^. For disability status, our results are consistent with studies that found a significantly higher percentage of people with disabilities to live near pollution sources^30,31^ and children with disabilities to reside in US school districts with greater air toxics exposure^32^. Although individuals aged 62 or more years were significantly underrepresented in neighborhoods with greater PM_2.5_ exposure, these results are similar to previous studies that found a higher proportion of elderly residents in areas with lower air pollution exposure^33,34^.

The second research objective focused on investigating whether PM_2.5_ concentration levels around PHDs were greater for those characterized by higher percentages of socially disadvantaged households. Multivariate GEEs revealed that PHDs with greater PM_2.5_ exposure contained significantly higher percentages of non-Hispanic Black, Hispanic, disabled, and extremely low-income households, after controlling for spatial clustering, urbanization level, and relevant characteristics of their host neighborhoods. The disproportionately large concentration of minority, disabled, and economically disadvantaged residents in PHDs most exposed to higher levels of outdoor PM_2.5_ pollution point to environmental and social injustices that go beyond our previous research finding regarding the significantly higher presence of PHD units and residents in more polluted neighborhoods.

Public housing in the US has been associated with poorer health outcomes, and the negative health experiences of PHD residents compared to non-PHD residents are well-documented^35–37^. Residents of federally assisted rental housing experience higher rates of asthma and other chronic diseases, and have lower access to health-promoting resources such as safe exercise spaces and healthy food outlets^38–41^. Evidence also indicates that health risks, such as pests, mold, combustion-related toxins, and inadequate ventilation, are considerably greater in subsidized rental housing developments compared to other rental housing^37,42^. These findings suggest that PHD residency in areas exposed to higher levels of outdoor air pollution could plausibly contribute to or worsen health outcomes for this marginalized group. In this context, the results of our study suggest that many PHD residents in the US are at a ‘multiple jeopardy’^43,44^ based on the convergence of their minority, disability, and poverty status, that is potentially amplified by the adverse effects of higher PM_2.5_ exposure at both their neighborhood and housing development locations.

While this study contributes important findings on exposure to air pollution for PHD residents, it is important to consider three limitations. First, our analyses are cross-sectional and cannot be used to explain the processes that led to the observed social disparities. Since longitudinal data was not used, the results cannot be used to infer the sequence of events that caused increased PM_2.5_ exposure for neighborhoods containing a significantly higher proportion of PHDs or PHDs with a higher proportion of socially disadvantaged households. More research is necessary to analyze policy decisions, as well as changing patterns of residential segregation, industrial and economic development, racial/ethnic migration, and other socioeconomic processes that are potentially responsible for the concentration of PHDs in areas exposed to greater PM_2.5_. However, our findings represent an important starting point for more detailed longitudinal analysis of the relationship between air pollution exposure and PHD residency.

Second, our study focuses specifically on federally subsidized rental housing developments associated with HUD’s public housing program, which included more residents (2.24 million) than any other type of HUD-assisted multifamily housing in 2015. Future research should consider extending this work to analyze PM_2.5_ exposure for properties affiliated with other HUD programs such as Section 202 (supportive housing for the elderly), Section 811 (supportive housing for persons with disabilities), and Section 8 project-based rental assistance, as well as other housing subsidy programs such as Community Development Block Grants, HOPE, and Indian Housing. We also did not examine subsidized rental housing affiliated with Section 8 Housing Choice Vouchers (HCVs), a program used by tenants to subsidize their rents in the private market housing of their choice. Although locations of households using Section 8 HCVs are not available in the HUD database utilized for this study, it is important to consider that more than 50% of residents receiving rental assistance from HUD used vouchers in the private housing market in 2015. Future research should explore additional or local data sources that can be used to evaluate air pollution exposure disparities for residents of tenant-based programs such as Section 8 HCVs.

Third, our study analyzed disparities associated with outdoor exposure to air pollution, but did not examine indoor air quality problems faced by public housing residents. A major impediment here is data availability since indoor air pollution estimates for PHDs are not currently available in the US. However, both outdoor and indoor pollution sources have been found to worsen indoor air quality in multifamily rental housing^45,46^. Residents of PHDs could be additionally burdened because households of lower socioeconomic status tend to spend more time at home due to lower employment levels^46^. Additionally, higher housing density in PHDs could lead to increased pollution exposure from neighboring housing units^46,47^. Recent research indicates while both housing characteristics (e.g., building quality, volume, and ventilation) and occupant behavior (e.g., smoking and cooking practices) contribute to indoor air pollution exposure, these risks vary across socioeconomic groups, with lower income households (e.g., those in PHDs) exposed to higher levels of indoor air pollution^35,46,47^.

In conclusion, our study reveals that public housing developments, units, and residents in the continental US are disproportionately located in neighborhoods with significantly higher outdoor PM_2.5_ concentrations. Within the population residing in PHDs, we also found that Black, Hispanic, disabled, and extremely low-income households are more likely to reside in developments around which PM_2.5_ exposure is significantly higher. Although this study did not examine PM_2.5_ pollution sources, our results could be potentially linked to previous assertions that a confluence of historical policies and practices have encouraged the construction of public housing in areas of environmental contamination and also allowed pollution sources such as hazardous industries to locate near these housing developments^23,24^. The findings of our study emphasize the growing need to analyze and reverse these patterns, as well as identify and address gaps in environmental, public health, and housing policies that place a significantly higher risk burden on public housing inhabitants.

## Materials and methods

### Fine particulate air pollution data

For assessing exposure to fine particulate air pollution, we use estimates of outdoor PM_2.5_ concentrations developed by the Center for Air, Climate and Energy Solutions (CACES), using national-scale empirical models as described in Kim et al.^25^. These models are based on publicly available concentration measurements from EPA regulatory monitors, and combine land use information and satellite-derived estimates of air pollution to predict concentrations for criteria air pollutants at multiple spatial scales, including census tracts and block groups. Census tracts are relatively permanent statistical subdivisions of a US county that generally encompass between 1500 and 8000 people, with an optimum size of 4000 people^48^. Tracts are designed to be fairly homogeneous with respect to the demographic and socioeconomic characteristics of the local population when they are first established. A census block group is a subdivision of a census tract that generally contains between 600 and 3000 people, with an optimum size of 1500 people^48^.

For this study, both census tract and block group level values of predicted PM_2.5_ concentrations were downloaded from the CACES Land Use Regression database for 2011 to 2015 (latest years available). While this database also provides ambient concentrations of other air pollutants (carbon monoxide, ozone, nitrogen dioxide, sulfur dioxide, and PM_10_), we focused specifically on PM_2.5_ because of its well-documented adverse health outcomes^1–10^ and higher exposure levels for socially disadvantaged groups in the US^13–21^.

For each census tract and block group in the continental US, the five-year average annual PM_2.5_ concentration was calculated as the arithmetic mean of five annual concentration values, from 2011 to 2015. This information was used to develop separate dependent variables to address each of our two research objectives. Tract-level mean values of PM_2.5_ concentration (2011–2015) were used for the tract-level analysis. For the PHD-level analysis, the average annual PM_2.5_ concentration (2011–2015) around each housing development was estimated using values from the block group in which their geographic coordinates were located. Specifically, we utilized a spatial join function in *ArcGIS Desktop 10.8.1* software that allocates selected attributes from a target polygon feature (i.e., PM_2.5_ concentration from a block group) to all point features located within the boundary of the target polygon (i.e., PHDs located inside or on the block group boundary).

### Public housing data

Since the US Housing Act of 1937, the US federal government has provided housing assistance to low-income renters under programs administered by the US Department of Housing and Urban Development (HUD). These HUD programs provide subsidies that reduce rents for lower income tenants who meet specific program eligibility requirements. Generally, households pay rent equaling 30% of their incomes, while the federal government pays the remainder of the rental costs. Public housing, the focus of this study, includes housing developments owned and operated by local Public Housing Authorities for which HUD provides capital and operating assistance^49^. In 2015, the total number of people residing in PHDs was about 2.24 million—higher than those living in any other type of HUD-assisted multifamily project-based rental housing.

Information on federally subsidized public housing for this study was derived from the online HUD *Picture of Subsidized Households*^26^. For each PHD, this database provides locational coordinates, number of occupied and unoccupied units, socio-demographic characteristics of resident households, and other relevant details. Our study was based on project level data for 2015 (compiled by HUD on December 31, 2015), to match our 2011–2015 annual average PM_2.5_ concentration values. Using *ArcGIS Desktop 10.8.1* software, the locations of 7000 PHDs (buildings) in the continental US were geocoded using latitude–longitude information. It should be noted that PHDs in the HUD database used for this study are depicted as a distinct address that is chosen to represent the general location of an entire PHD. When a PHD comprises multiple buildings, the building with the largest number of units is selected to represent the location of the development.

In the *Picture of Subsidized Households* database, HUD suppresses data for count variables with values smaller than 11 (coded as − 4) to preserve confidentiality and data for specific variables are not reported (coded as − 5) when reporting rates for the PHD are smaller than 50%. To address suppressed data on the number of units and resident population in 311 of the 7,000 PHDs in the continental US, we substituted a value of 1 to replace values of − 4 and − 5. This is likely to undercount the actual number of units and residents in these 311 PHDs, but provides a conservative non-zero estimate for our census tract-level analysis^50^.

### Variables

The first phase of our study sought to examine whether PHD locations, units, and residents, respectively, are overrepresented in census tracts with greater PM_2.5_ exposure, after controlling for spatial clustering and relevant socio-demographic factors. This necessitated the construction of three different independent variables to assess the public housing status of each tract. The first variable indicated the presence or absence of PHDs in the tract, a dichotomous measure coded as either 1 or 0. The second and third variables are continuous and estimate the number of PHD units and residents, respectively, expressed as a percentage of the total number of all housing units and population in each tract from the 2015 American Community Survey (ACS) five-year estimates. We excluded tracts with low population (< 500) and housing (< 200) counts to ensure stable proportions for our variables, as well as tracts located in counties without PHDs. This led to the removal of 11,146 (15.4%) of 72,263 tracts in the continental US. The first phase of our analysis thus encompasses a total of 61,117 tracts with at least 500 people and 200 housing units, from the 1,892 counties that contained PHDs in 2015.

To control for tract-level socio-demographic characteristics, we used variables from the 2015 ACS five-year estimates that have been found to be significantly related to PM_2.5_ exposure in previous national-scale US studies^14,15,17–19,21,27^ and are comparable to variables available in the HUD dataset on public housing utilized for PHD-level analysis. To examine the effects of ethnicity and race, we included the percentages of individuals who identified themselves as Hispanic or Latino (of any race) and non-Hispanic Black alone—the two largest ethnic/racial minority groups in the US. We combined the percentages of non-Hispanic American Indian/Alaska Native alone, non-Hispanic Asian alone, non-Hispanic Pacific Islander/Native Hawaiian alone, non-Hispanic another race, and multiple races into a single category that represents non-Hispanic other non-White (minority) individuals. We excluded the percentage of non-Hispanic White residents from our multivariable models to ensure that results for the minority ethnic/racial groups are interpretable relative to this category. The percentage of people with an annual income below the poverty level was used to represent socioeconomic status. Additional measures of social disadvantage comprised the percentage of people with a disability, those aged 62 or more years, and female-headed households. The disability variable in the ACS encompasses civilian non-institutionalized individuals reporting at least one of these difficulties: hearing, vision, cognitive, ambulatory, self-care, and independent living. In addition to these socio-demographic characteristics, three control variables were included in our multivariable models. To account for urban–rural differences, we used Rural–Urban Commuting Area (RUCA) codes developed by the US Department of Agriculture that classify census tracts using measures of population density, urbanization, and daily commuting^51^. We created two dummy variables to identify tracts located in metropolitan (RUCA codes 1–3) and micropolitan areas (RUCA codes 4–6), respectively. We excluded RUCA codes 7–10 (small towns and rural areas) from our models, making them the interpretive reference for variables representing the metropolitan and micropolitan categories. Since PHDs are classified as multifamily housing, we included the percentage of housing units in the tract that are single-family detached homes to adjust for the prevalence of non-PHD multifamily housing.

The second phase of our study focused on determining if the average PM_2.5_ concentration (2011–2015) in census block groups hosting PHDs was greater for PHDs containing higher percentages of socially disadvantaged households. This phase encompasses a total of 6,685 PHDs with at least 11 residents for which detailed socio-demographic data on resident household characteristics were available (no missing data for included variables) in the HUD *Picture of Subsidized Households* database for December 31, 2015^13^. For ethnic/racial minority status, we included the percentages of households where the head of the household was Hispanic and non-Hispanic Black. Information on the percentage of households belonging to other non-Hispanic minority categories (e.g., non-Hispanic Asian, Native American, and Other Race) were combined into a single variable. Our analysis included three additional indicators of social disadvantage that align closely with the tract-level independent variables used in Phase 1: the percentage of female-headed households, those in which the eldest of the household head or spouse was aged 62 or older, and residents with a disability. To measure socioeconomic status, we used the percentage of extremely low-income households (HUD definition), as described in Table 3. We also used the same set of control variables utilized for our tract-level analysis in the previous phase: metropolitan or micropolitan status of the tract hosting the PHD based on RUCA codes, and percentage of single-family housing units to adjust for the prevalence of non-PHD multifamily housing.

### Statistical models

For the first phase of analysis, three multivariable models were constructed to independently examine the association of the presence of PHDs, percentage of PHD units, and percentage of population residing in PHD units, respectively, with average tract-level PM_2.5_ concentrations, after controlling for relevant socio-demographic variables and spatial clustering. In the second phase, a single multivariable model was constructed to examine the relationship between average PM_2.5_ concentrations in the census block group hosting the PHD and selected socio-demographic characteristics of PHDs, after controlling for a set of variables similar to those used in Phase 1. Our multivariable models for both phases are based on generalized estimating equations (GEEs) with robust (i.e., Huber/White) covariance estimates, which extend the generalized linear model to accommodate clustered data^52^. GEEs relax several assumptions of traditional regression models and impose no strict distributional assumptions for the variables analyzed (e.g., normality), while accounting for clustering of analytic units. Recent studies indicate that GEEs based on theoretically informed clusters are more advantageous for analyzing social disparities in the distribution of environmental hazards compared to other techniques such as spatial autoregressive models, mixed models with random effects, and multilevel modeling approaches^17,50,53^. To estimate a GEE model, clusters of observations are defined based on the assumption that observations from within a cluster are correlated, whereas observations from different clusters are independent^54^. The cluster definition for the tract-level analysis was based on the US county within which the tract is located by median decade of housing construction, which ranged from “2010 or later” to “before 1939”. This definition corresponds to historical–geographical formation of environmental inequalities^55^ and is consistent with previous national-scale US studies on social disparities in air pollution^17,56^. This combination of county (1,892) and median decade of housing stock (10) for each tract yielded a total of 7,492 clusters for the tract-level GEEs. Clusters were defined for the PHD-level analysis using the city in which the building was located, since federally subsidized rental housing within a particular city can be expected have to have certain similarities compared to those located elsewhere^46^. This resulted in a total of 3337 clusters of PHDs in the PHD-level GEE model.

An intra-cluster dependency correlation matrix also needs to be specified for estimating a GEE^54^. After experimentation with several different specifications, the ‘independent’ correlation matrix was chosen for the three GEEs in Phase 1 and the ‘exchangeable’ correlation matrix was selected for the GEE in Phase 2. We selected these specifications because they yielded the best statistical fit based on the QIC (quasi-likelihood under the independence model criterion). To further improve model fit, several different distributions (e.g., normal, gamma, and inverse Gaussian) appropriate for non-zero continuous dependent variables were tested with identity and logarithmic link functions. An identity link function models relationships between the dependent and independent variables linearly, while a log link function represents natural logarithmic relationships. The gamma distribution with identity link function was chosen for all GEEs because this function indicated the best fit based on the QIC.

All independent variables were standardized, and standardized coefficients are provided in tables summarizing the GEE results. Two-tailed *p* values from the Wald Chi-Square test were used to test the statistical significance of each variable coefficient. Potential multicollinearity among these variables were also examined using the multicollinearity condition index for the combination of all independent variables included in each GEE. This multicollinearity condition index was smaller than 10.0 in all multivariable models, confirming that the GEEs are not affected by multicollinearity. We also tested the residuals from our four GEE models for spatial autocorrelation using a contiguity-based (queen) spatial weights matrix. Non-significant (*p* > 0.05) values of the Moran’s *I* statistic indicated that spatial autocorrelation did not affect our statistical model results.

## Data Availability

Publicly available data from the CACES, HUD, and ACS were used for this study, as described in the Materials and methods section of this paper. Additional data related to this paper may be requested from the authors.
